# Predictors of the Efficacy for Daytime Sleepiness in Patients With Obstructive Sleep Apnea With Continual Positive Airway Pressure Therapy: A Meta-Analysis of Randomized Controlled Trials

**DOI:** 10.3389/fneur.2022.911996

**Published:** 2022-06-27

**Authors:** Zhiqiang Li, Sijie Cai, Jing Wang, Rui Chen

**Affiliations:** ^1^Department of Respiratory and Critical Care Medicine, Sleep Center, The Second Affiliated Hospital of Soochow University, Soochow University, Suzhou, China; ^2^Department of Pulmonary and Critical Care Medicine Affiliated Kunshan Hospital of Jiangsu University, Suzhou, China

**Keywords:** obstructive sleep apnea, continuous positive airway pressure, excessive daytime sleepiness, predictors, efficacy

## Abstract

**Objective:**

The main aim of this meta-analysis was to evaluate the predictors of the efficacy of continuous positive airway pressure (CPAP) in ameliorating excessive daytime sleepiness (EDS) in patients with obstructive sleep apnea (OSA).

**Methods:**

Randomized controlled trials (RCTs) published between January 1994 and October 2021 were searched in the PubMed, EMBASE, and Cochrane Library databases. The weighted mean differences (WMDs) for the Epworth Sleepiness Scale (ESS) scores, the Multiple Sleep Latency Test (MSLT), and the Maintenance of Wakefulness Test (MWT) were pooled in STATA.

**Results:**

A total of 41 RCTs involving 7,332 patients were included. CPAP therapy was found to be significantly associated with changes in ESS (WMD = −2.14, *P* < 0.001), MSLT (WMD = 1.23, *P* < 0.001), and MWT (WMD = 1.6, *P* < 0.001). Meta-regression analysis and subgroup analysis indicated that in mild OSA, the efficacy of CPAP therapy for subjective EDS was limited to patients <50 years of age, with a baseline body mass index (BMI) of ≥30 kg/m^2^, baseline ESS score of ≥11, therapy adherence for ≥3 h/night, and treatment duration of ≥2 months. In moderate OSA, significant differences were observed in the changes in ESS among groups stratified by baseline ESS score (*P* = 0.005), adherence (*P* < 0.001), treatment duration (*P* = 0.009), and trial design type (*P* = 0.001). In severe OSA, this difference was observed among groups stratified by baseline BMI (*P* = 0.0*2*8), baseline ESS score (*P* = 0.001), and adherence (*P* = 0.047). Patients with moderate-severe OSA but not mild OSA showed significant improvements in MSLT. Patients with the age <50 years or BMI ≥33 kg/m^2^ had a more significant increase in MWT.

**Conclusion:**

Continuous positive airway pressure therapy improved subjective and objective sleepiness in patients with OSA. Age, baseline BMI, baseline ESS score, adherence, and duration of treatment may predict the effects of CPAP on EDS in patients with OSA. Notably, the baseline ESS scores and adherence were stable predictors regardless of OSA severity.

## Introduction

Obstructive sleep apnea (OSA) is among the most common sleep breathing disorders and is characterized by repeated obstruction of the upper airway during sleep, thus causing sleep fragmentation and intermittent hypoxia (1). Excessive daytime sleepiness (EDS), the most frequently self-reported symptom of OSA, may negatively affect mood, cognitive ability, and quality of life (2). EDS has been reported to affect 40.5%−58% of patients with OSA and is a risk factor for motor vehicle accidents (3). In recent years, EDS has received increased attention in the field of sleep medicine, and several clinical assessment tools are now available for EDS evaluation, including objective daytime sleepiness based on the multiple sleep latency test (MSLT) (4), the maintenance of wakefulness test (MWT) (5), and the subjective daytime sleepiness measured with the Epworth Sleepiness Scale (ESS) (6).

Continuous positive airway pressure (CPAP) therapy is the main treatment for OSA, particularly in moderate to severe cases (7). Previous meta-analyses have demonstrated that CPAP significantly decreases subjective but not objective sleepiness in patients with OSA (8, 9). Although EDS can be decreased with CPAP therapy, a substantial number of patients still experience EDS after treatment. Between 9 and 22% of patients still experience residual EDS after CPAP treatment (10). The efficacy of CPAP therapy for EDS is inconsistent due to several potential confounding factors, including heterogeneity in age, obesity, adherence, duration of CPAP therapy, and OSA severity. Given the heterogeneity and complexity of OSA, accurate CPAP therapy should involve a combination of several dimensions, including demographic characteristics, appropriate CPAP protocols, and pathophysiology. Therefore, clinical factors associated with CPAP efficacy regarding daytime sleepiness in patients with OSA must be identified. The purpose of this meta-analysis was to explore the relationship between different clinical subtypes of OSA and the efficacy of CPAP therapy for EDS.

## Methods

### Literature Search

We conducted a literature search of the PubMed, EMBASE, and Cochrane Library databases for articles published between January 1994 and October 2021. Combinations of Medical Subject Heading (MeSH) terms and free-text words were searched according to the Population, Intervention, Control, Outcome, and Study Design (PICOS) principle. Search terms for the population category were sleep apnea, obstructive (MeSH) OR (OSA syndrome) OR (OSA) OR (OSAHS) OR (sleep apnea-hypopnea syndrome) OR (syndrome, OSA) OR (upper airway resistance sleep apnea syndrome) OR (syndrome, upper airway resistance, and sleep apnea). Search terms for the intervention category were continuous positive airway pressure (MeSH) OR (CPAP ventilation) OR (nasal CPAP) OR (nCPAP ventilation) OR (biphasic CPAP) OR [bi-level positive airway pressure (BiPAP)] OR (bilevel CPAP). Search terms for the control category were (oral placebo) OR (sham CPAP) OR (placebo CPAP) OR (conservative treatment). Search terms for the outcome category were (the ESS) OR (subjective sleepiness) OR (the MSLT) OR (objective sleepiness) OR (the MWT) OR (objective wakefulness). Search terms for the study design category were (randomized controlled trial) OR (randomized) OR (placebo).

### Inclusion and Exclusion Criteria

The inclusion criteria were as follows: (1) study design: randomized controlled trials for CPAP therapy vs. sham control; (2) participants: OSA diagnosed with an apnea-hypopnea index (AHI) ≥5 events/h on the basis of polysomnography; (3) study reported outcomes: assessment of at least one of the sleepiness indicators, ESS, MSLT, or MWT. The exclusion criteria were as follows: repeat studies, abstracts, case reports, reviews, letters, studies with invalid data, and patients <18 years of age.

### Quality Assessment

The JADAD scale (11) and Cochrane risk bias assessment tools (12) were used to evaluate the quality of RCTs and were independently completed and verified by two researchers. A modified JADAD score of 4–7 represented high-quality research and 1–3 signified low-quality research. All literature included in the meta-analysis was scored 4–7, indicative of high-quality research (Table 1). The Cochrane risk bias assessment tool (Supplementary Figure 1) was applied to evaluate seven important sources of bias (random sequence generation, allocation concealment, blindness of subjects and researchers, blindness of outcome evaluation, incomplete data, selective reporting of results, and other biases). Green color represents low risk, yellow color represents medium risk, and red color represents high risk.

**Table 1:** Basic characteristics of patients and studies included in the meta-analysis.

**Study**	***n* (E/C)**	**Experimental group**	**Control group**	**Treatment** **duration**	**Trail design**	**Age (years)**	**BMI (kg/m^**2**^)**	**AHI (events/h)**	**ESS**	**Compliance (h/night)**	**Comorbidity**	**Jadad**
Engleman et al. (13)	32 (17/15)	CPAP	Oral placebo	4 weeks	Crossover	49	33	28	NR	3.4	No	4
Engleman et al. (14)	16 (8/8)	CPAP	Oral placebo	4 weeks	Crossover	52	29.8	11	14	2.8	No	4
Redline et al. (15)	97 (51/46)	CPAP	conservative	8 weeks	Parallel	48.1	33.4	14.6	10.4	3.1	No	4
Engleman et al. (16)	23 (12/11)	CPAP	Oral placebo	4 weeks	crossover	47	30	43	12	2.8	No	4
Engleman et al. (17)	34 (17/17)	CPAP	Oral placebo	4 weeks	crossover	44	30	10	13	2.8	No	4
Ballester et al. (18)	105 (68/37)	CPAP	conservative	3 months	Parallel	53	32	56	12	5.2	No	3
Faccenda et al. (19)	68 (33/35)	CPAP	placebo	4 weeks	Parallel	50	30	35	15	3.3	No	4
Barbe et al. (20)	54 (29/25)	CPAP	Sham CPAP	6 weeks	Parallel	54	29	54	7	4.5	No	6
Montserrat et al. (21)	55 (23/22)	CPAP	Sham CPAP	6 weeks	Parallel	55.65	30.31	50.52	16.13	4.3	No	4
Monasterio et al. (22)	125 (66/59)	CPAP	conservative	6 months	Parallel	53	29.4	20	12.1	4.8	No	4
Barnes et al. (23)	28 (14/14)	CPAP	Oral placebo	8 weeks	crossover	45.5	30.2	12.9	11.2	3.5	No	6
Becker et al. (24)	32 (16/16)	CPAP	Subtherapeutic	9 weeks	Parallel	54.4	33.3	62.5	14.4	5.5	No	5
Woodson et al. (25)	54 (26/28)	CPAP	placebo	8 weeks	Parallel	51.7	29.1	19.8	12.6	4.2	No	4
Pelletier-Fleury et al. (26)	171 (82/89)	CPAP	No treatment	6 months	Parallel	53.8	30.5	53.2	10.6	5.4	No	4
Barnes et al. (27)	80 (40/40)	CPAP	Oral placebo	3 months	crossover	46.4	30	21.5	10.2	3.6	No	6
Marshall et al. (28)	29 (15/14)	CPAP	Sham CPAP	3 weeks	crossover	50.5	31.5	21.6	12.5	4.9	No	6
Hui et al. (29)	56 (28/28)	CPAP	Subtherapeutic	12 weeks	Parallel	50.8	27	31.2	11.1	5.1	No	4
Lam et al. (30)	67 (34/33)	CPAP	conservative	10 weeks	Parallel	47	27.6	23.8	12	4.4	No	4
Coughlin et al. (31)	46 (23/23)	CPAP	Subtherapeutic	6 weeks	crossover	49	36.1	39.7	13.8	3.9	No	4
West et al. (32)	42 (20/22)	CPAP	placebo	3 months	Parallel	57.8	36.6	NR	14.7	3.6	Type 2 diabetes	5
Kohler et al. (33)	102 (51/51)	CPAP	Subtherapeutic	4 weeks	Parallel	48.1	35.8	41.9	15.8	4.7	No	5
West et al. (34)	36 (16/20)	CPAP	placebo CPAP	3 months	Parallel	57.2	37.4	13.4	3.8	3.8	Type 2 diabetes	4
Barbe et al. (35)	374 (191/183)	CPAP	conservative	12 months	Parallel	56	33	49	6.4	4.2	Hyptension	5
Duran-Cantolla et al. (36)	340 (169/171)	CPAP	sham	3 months	Parallel	53.2	31.9	44.5	10.3	4.5	Hyptension	6
Tomfohr et al. (37)	59 (29/30)	CPAP	Oral placebo	3 weeks	Parallel	48.14	30.57	38.64	9.26	5.5	No	4
Ryan et al. (38)	44 (22/22)	CPAP	standard rehabilitation	4 weeks	Parallel	60.28	28.8	38.5	4.4	5	Stoke	5
Phillips et al. (39)	37 (18/19)	CPAP	placebo	2 months	crossover	49	32.1	41.2	11.2	4.5	No	6
Sivam et al. (40)	27 (14/13)	CPAP	sham	2 months	crossover	47.3	31.3	37.2	10	4.6	No	5
Amaro et al. (41)	12 (6/6)	CPAP	Nasal dilator strips	12 months	crossover	52	33.5	38	12	6.6	No	4
Weaver et al. (42)	223 (113/110)	CPAP	sham	8 weeks	crossover	49.5	33.2	12.8	15.21	4	No	4
Kushida et al. (43)	1,098 (556/542)	CPAP	Sham CPAP	6 months	Parallel	52.2	32.4	39.7	10.07	4.2	No	4
Martinez-Garcia et al. (44)	194 (98/96)	CPAP	usaul-care	12 weeks	Parallel	57.8	34.3	41.3	8.9	5	Hyptension	6
McMillan et al. (45)	278(138/140)	CPAP	BSC	12 months	Parallel	70.9	33.9	29.4	11.6	2.4	No	4
Martinez-Garcia et al. (46)	224 (115/109)	CPAP	No treatment	3 months	Parallel	75.4	33	53.5	9.6	4.9	No	4
Dalmases et al. (47)	33(17/16)	CPAP	conservative	3 months	Parallel	70.76	29.94	61.16	7.94	6	No	4
Salord et al. (48)	80 (42/38)	CPAP	conservative	12 weeks	Parallel	48.5	45.7	68.3	7.9	5.4	No	4
Joyeux-Faure et al. (49)	36 (18/18)	CPAP	Sham CPAP	6 weeks	Parallel	54.9	29.6	41.5	10.4	4.5	No	4
McEvoy et al. (50)	2,707 (1,366/1,341)	CPAP	Usual-Care	3.7 years	Parallel	61.3	28.8	29	7.3	3.3	Cardiovascular disease	4
Zhao et al. (51)	138 (68/70)	CPAP	conservative	12 months	Parallel	63.8	31.1	26.2	8	3.44	No	4
Gaisl et al. (52)	52 (26/26)	CPAP	Sham CPAP	2 weeks	Parallel	60.1	33	38.8	12.1	3.3	No	5
Baillieul et al. (53)	24 (12/12)	CPAP	Sham CPAP	8 weeks	Parallel	60.4	28	54.4	11	4.7	No	4

*N(E/C), number of patients (experimental group/control group); BMI, body mass index; AHI, apnea-hypopnea index; ESS, Epworth Sleepiness Scale; NR, not reported*.

### Statistical Analyses

This meta-analysis was performed according to the PRISMA guidelines (54). The mean differences and standard errors in treatment efficacy between the CPAP and control groups were pooled in Stata 12.0. Heterogeneity between studies was assessed with *Q*-tests, and the *I*^2^-value indicated the degree of heterogeneity among therapeutic effects. Substantial heterogeneity between trials was indicated by *P* < 0.1 and *I*^2^ > 50%. A two-tailed test was selected (α = 0.05). Meta-regression and subgroup analyses were used to explore potential sources of heterogeneity. Meta-regression models were performed to assess changes in the efficacy of CPAP, including the effects of basic study and patient characteristics, such as RCT design type (parallel or crossover), treatment duration, age, BMI, AHI, ESS scores, adherence, and comorbidity. Begg's and Egger's tests were used to quantitatively evaluate publication bias, and a *P*-value > 0.5 was considered to indicate no publication bias. Sensitivity analyses were performed by individually eliminating each study and recalculating the pooled weighted mean difference (WMD) and 95% CI to determine the reliability of the results.

## Results

### Literature Selection

**Figure 1:**
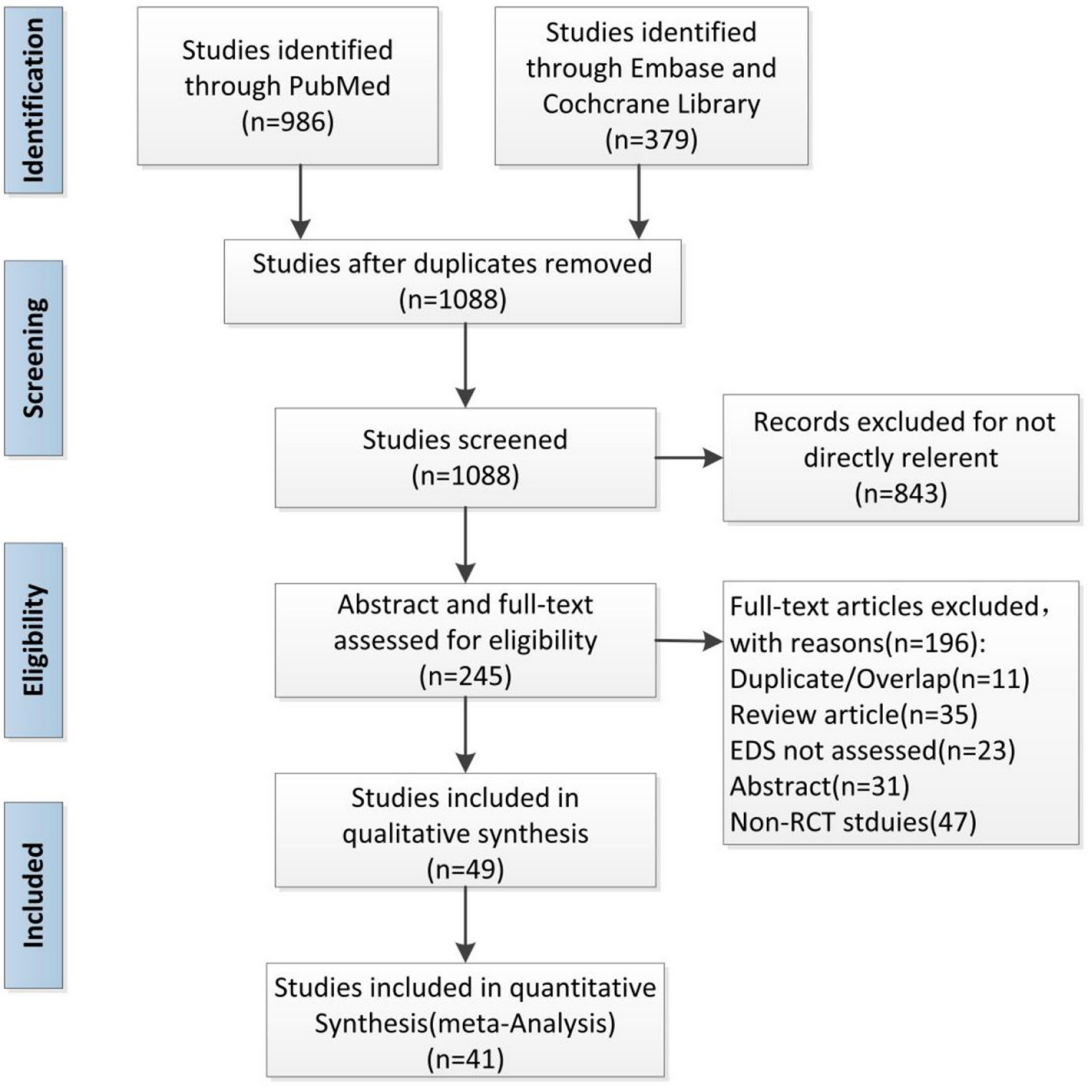
Screening process for study selection and inclusion.

A total of 1,365 articles were retrieved by independent assessors, with the substantive agreement. Overall, 267 duplicate publications were excluded. The remaining 245 RCTs were obtained after the removal of 843 irrelevant articles. We excluded 196 studies according to the inclusion and exclusion criteria and removed eight articles with invalid data. Finally, 41 RCTs, representing 7,332 patients, were integrated into our meta-analysis (Figure 1).

### Data Extraction and Quality Assessment

Baseline characteristics of the included studies and patients are shown in Table 1. The first author, year of publication, number of patients, intervention and control groups, RCT design, treatment duration, baseline age, BMI, AHI, ESS score, adherence, comorbidity, and outcome data (ESS/MSLT/MWT) were extracted. The studies included 29 parallel RCTs and 12 crossover RCTs. The treatment duration lasted from 1 month to 3.7 years. The patients' baseline age, BMI, AHI, ESS score, and adherence were 44.0–75.4 years, 27.2–45.7 kg/m^2^, 10.0–68.3 events/h, 4.4–15.8 points, and 2.8–6.6 h/night, respectively. Engleman et al. (13) did not report baseline ESS scores, and West 2007 (32) did not report baseline AHI. Forty studies reported ESS (14–53), eight studies reported MSLT (13–17, 20, 22, 23), and six studies reported MWT (17, 27, 28, 32, 34, 43).

### Overall Pooled Effect Sizes of ESS, MSLT, and MWT (Meta-Analysis of CPAP for Ameliorated Sleepiness)

**Figure 2:**
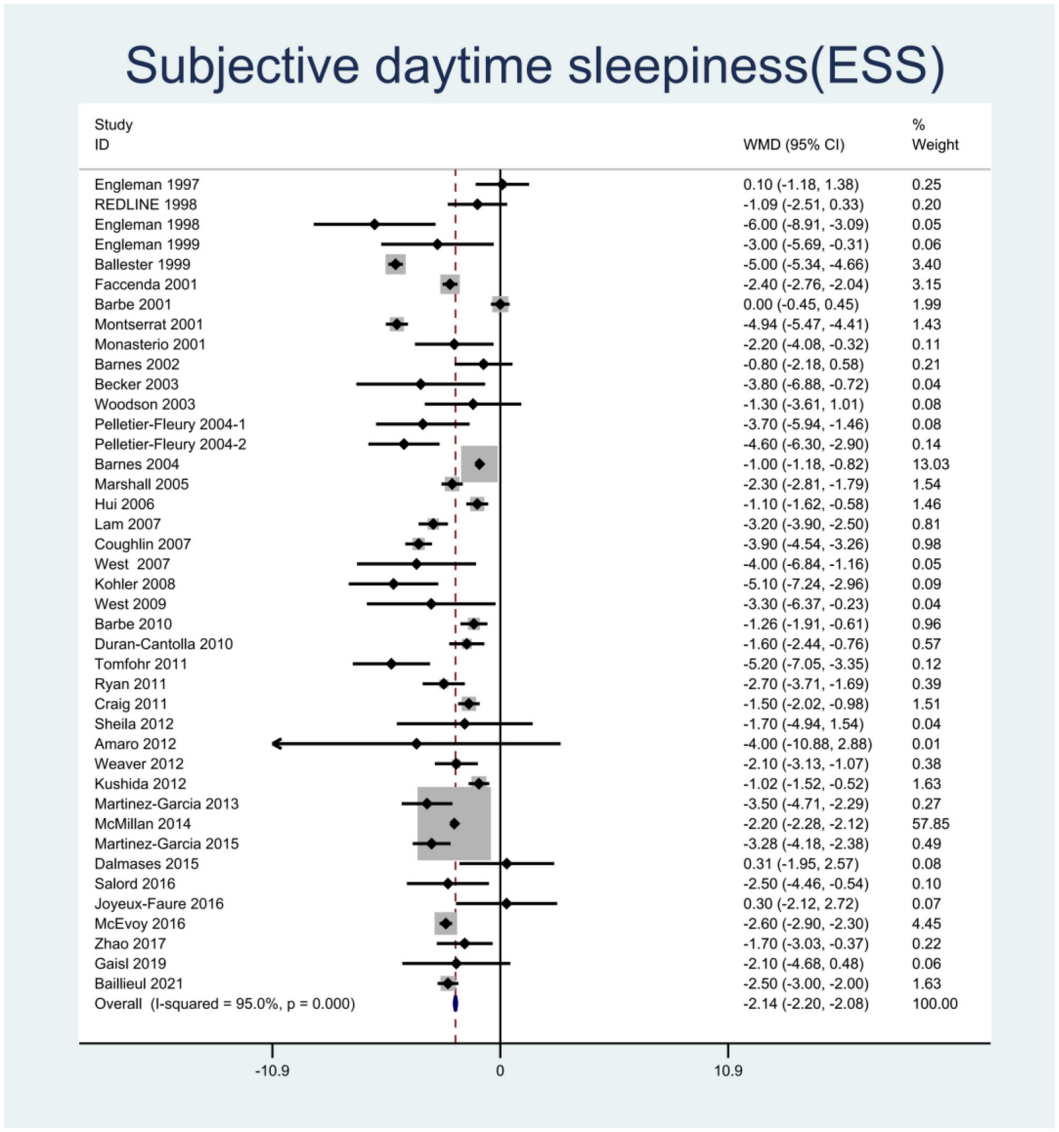
Forest plot showing pooled weighted mean difference (WMD) for Epworth Sleepiness Scale (ESS) in studies on continuous positive airway pressure (CPAP), compared with control group.

**Figure 3:**
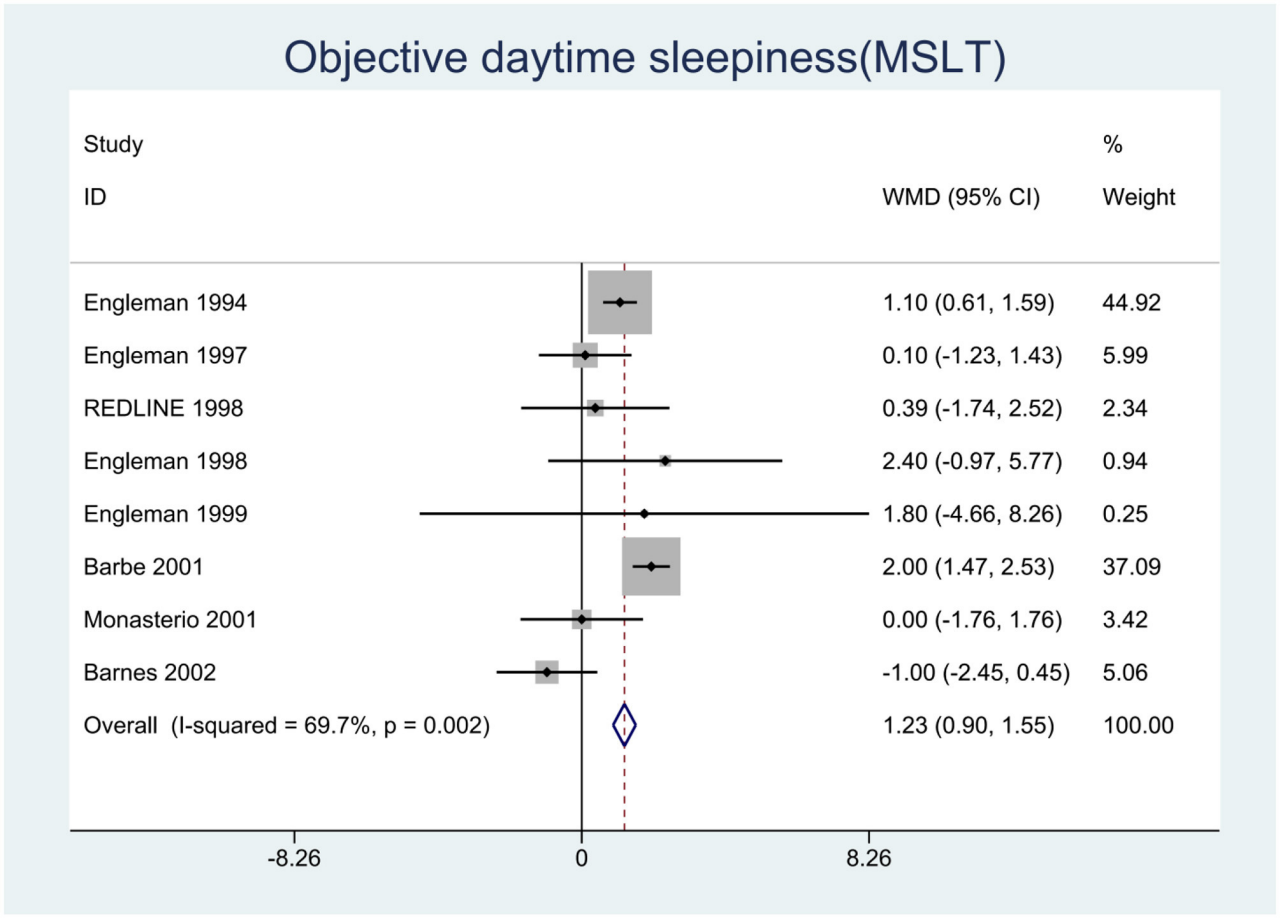
Forest plot showing pooled weighted mean difference (WMD) for Multiple Sleep Latency Test (MSLT) in studies on continuous positive airway pressure (CPAP), compared with control group.

**Figure 4:**
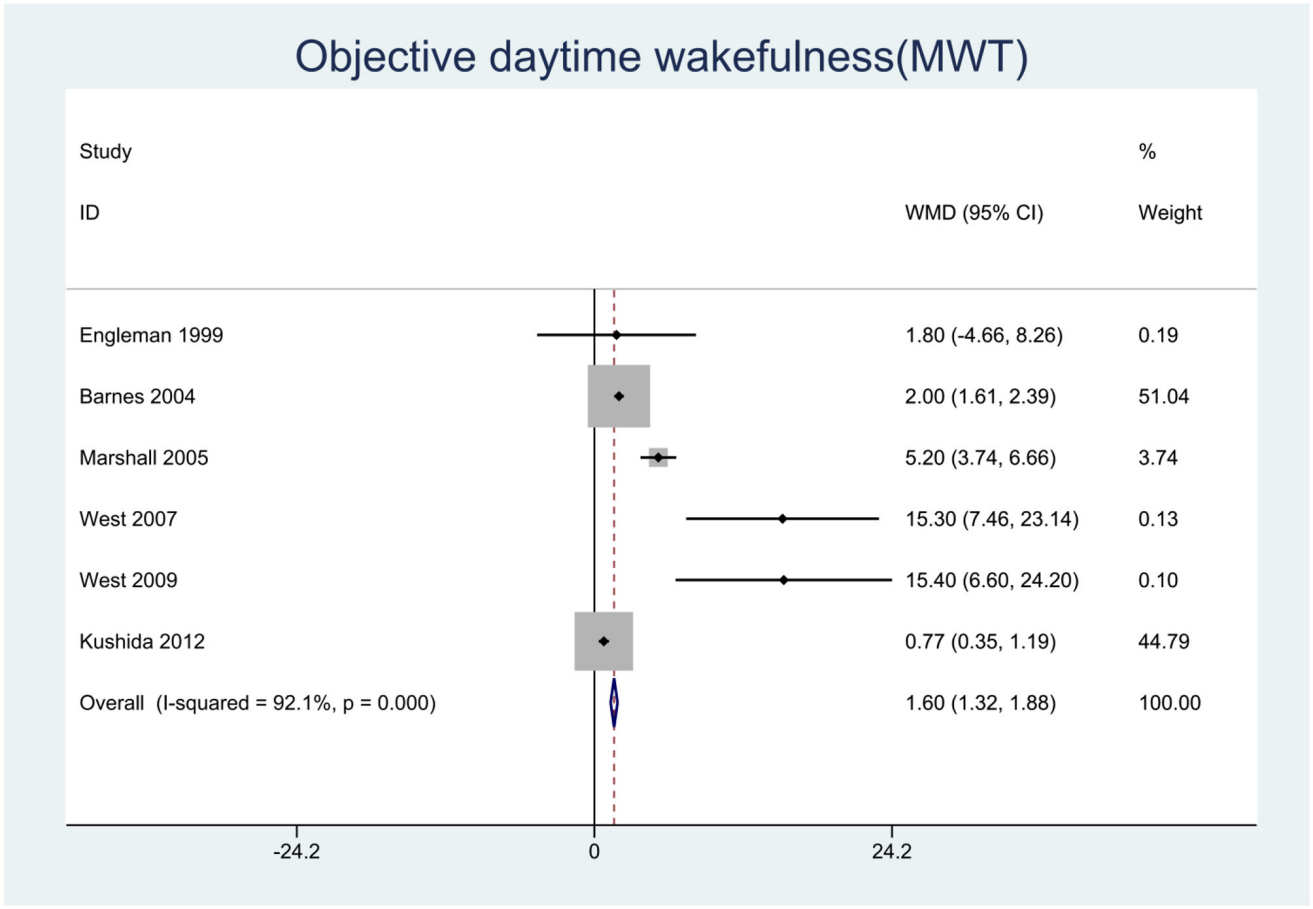
Forest plot showing pooled weighted mean difference (WMD) for Maintenance of Wakefulness Test (MWT) in studies on continuous positive airway pressure (CPAP), compared with control group.

We pooled the effect sizes to assess the effects of CPAP therapy on sleepiness. After controlling for the placebo effect, CPAP treatment was found to decrease the ESS scores by 2.14 points (95% CI: −2.20 to −2.08, *P* < 0.001), *I*^2^ = 95% (Figure 2), prolong MSLT by 1.23 min (95% CI: 0.90 to 1.55, *P* < 0.001), *I*^2^ = 69.7% (Figure 3), and increase MWT by 1.6 min (95% CI: 1.32 to 1.88, *P* < 0.001), *I*^2^ = 92.1% (Figure 4). The severity of OSA was classified as mild (5 ≤ AHI <15 events/h), moderate (15 ≤ AHI <30 events/h), or severe (AHI ≥ 30 events/h). In mild, moderate, and severe OSA, the ESS scores decreased by 1.29 points (95% CI: −1.89 to −0.70, *P* < 0.001), *I*^2^ = 53.2%, 2.03 points (95% CI: −2.11 to −1.96, *P* < 0.001), *I*^2^ = 95.5%, and 2.58 points (95% CI: −2.72 to −2.45, *P* < 0.001), *I*^2^ = 95.7%, respectively.

### Meta-Regression and Subgroup Analysis for Heterogeneity in the Change in Subjective EDS

The results of ESS scores in mild OSA are summarized in Table 2 and Supplementary Figure 4. Meta-regression demonstrated that the efficacy of CPAP therapy was significantly associated with BMI (segmentation point of BMI: 30 kg/m^2^) (*P* = 0.007) and adherence (*P* = 0.0*2*9) after adjustment for age. Subgroup analysis indicated that the efficacy of CPAP therapy was limited to patients <50 years of age (<50: WMD = −1.6, *P* < 0.001 vs. ≥50: WMD = −0.4, *P* = 0.505), with BMI ≥30 kg/m^2^ (<30: WMD = 0.1, *P* = 0.878 vs. ≥30: WMD = −1.68, *P* < 0.001), ESS scores ≥11 points (<11: WMD = −1.09, *P* = 0.13*2* vs. ≥11: WMD = −1.34, *P* < 0.001), adherence ≥3 h/night (<3 h: WMD = −0.47, *P* = 0.4*2*4 vs. ≥3 h: WMD = −1.59, *P* < 0.001), and treatment duration = 2–3 months (1 month: −0.47, *P* = 0.4*2*4 vs. 2 months: WMD = −1.5, *P* < 0.001 vs. 3 months: WMD = −3.3, *P* = 0.035).

**Table 2:** Meta-regression and subgroup analysis for subjective excessive daytime sleepiness (EDS) based on Basic characteristics of patients and studies in different severity of OSA.

**ESS variables**	**Numbers of study**	**Subgroup analysis**			**Meta regression**
		**WMD**	**95% CI**	***P*-Vaue**	**Interaction *P***
**5** **≤AHI** **<** **15 events/h**	6				
Baseline age					
<50	4	−1.60	−2.29, −0.91	<0.001	0.313
≥50	2	−0.40	−1.58,0.78	0.505	
Baseline BMI					
<30	1	0.10	−1.18,1.38	0.878	0.007
≥30	5	−1.68	−2.35, −1.01	<0.001	
Baseline ESS score					
<11	1	−1.09	−2.51,0.33	0.132	0.58
≥11	5	−1.34	−1.99, −0.68	<0.001	
Compliance					
<3 h	2	−0.47	−1.63,0.68	0.424	0.029
≥3 h	4	−1.59	−2.29, −0.90	<0.001	
<4 h	5	−0.89	−1.62, −0.16	0.017	0.138
≥4 h	1	−1.29	−1.89, −0.70	<0.001	
Treatment duration					
1 month	2	−0.47	−1.63,0.68	0.424	0.159
2 months	3	−1.50	−2.21, −0.79	<0.001	
3 months	1	−3.30	−6.37, −0.22	0.035	
Trail design					
Parallel	2	−1.48	−2.77, −0.19	0.025	0.674
crossover	4	−1.24	−1.91, −0.57	<0.001	
Comorbidity					
Yes	1	−3.30	−6.37, −0.22	0.035	0.256
No	5	−1.20	−1.82, −0.61	<0.001	
**15** **≤AHI** **<** **30 events/h**	9				
Baseline age					
<50	2	−1.13	−1.30, −0.96	<0.001	0.617
≥50	7	−2.23	−2.31, −2.15	<0.001	
Baseline BMI					
<30	3	−2.67	−2.95, −2.40	<0.001	0.117
≥30	6	−1.99	−2.06, −1.91	<0.001	
Baseline ESS score					
<11	4	−1.42	−1.57, −1.27	<0.001	0.005
≥11	5	−2.22	−2.30, −2.13	<0.001	
Compliance					
<3 h	1	−2.20	−2.28, −2.12	<0.001	0.986
≥3 h	8	−1.56	−1.70, −1.42	<0.001	
<4 h	4	−2.01	−2.09, −1.94	<0.001	<0.001
≥4 h	5	−2.59	−2.98, −2.20	<0.001	
<5 h	8	−2.03	−2.10, −1.96	<0.001	0.278
≥5 h	1	−3.70	−5.94, −1.46	0.001	
Treatment duration					
<3 month	3	−2.57	−2.98, −2.16	<0.001	0.009
≥3 months	6	−2.02	−2.09, −1.94	<0.001	
Trail design					
Parallel	7	−2.24	−2.32, −2.16	<0.001	0.001
crossover	2	−1.14	−1.30, −0.97	<0.001	
Comorbidity					
Yes	1	−2.6	−2.90, −2.30	<0.001	0.793
No	8	−2.0	−2.07, −1.93	<0.001	
**AHI** **≥30 events/h**	25				
Baseline age					
<50	7	−2.69	−3.06, −2.32	<0.001	0.122
≥50	18	−2.57	−2.72, −2.42	<0.001	
Baseline BMI					
<30	6	−1.17	−1.44, −0.90	<0.001	0.028
≥30	19	−3.08	−3.24, −2.92	<0.001	
Baseline ESS score					
<11	13	−1.30	−1.55, −1.06	<0.001	0.001
≥11	12	−3.22	−3.39, −3.05	<0.001	
Compliance					
<3 h	1	−6.00	−8.91, −3.09	<0.001	0.109
≥3 h	24	−2.58	−2.72, −2.45	<0.001	
<4 h	4	−2.78	−3.09, −2.48	<0.001	0.904
≥4 h	21	−2.53	−2.69, −2.38	<0.001	
<5 h	15	−2.12	−2.29, −1.96	<0.001	0.047
≥5h	10	−3.71	−3.97, −3.45	<0.001	
Treatment duration					
<3 month	14	−2.34	−2.57, −2.20	<0.001	0.487
≥3 months	11	−2.84	−3.05, −2.63	<0.001	
Trail design					
Parallel	20	−2.58	−2.72, −2.45	<0.001	0.832
Crossover	5	−2.51	−2.90, −2.11	<0.001	
Comorbidity					
Yes	4	−1.89	−2.31, −1.46	<0.001	0.557
No	21	−2.67	−2.82, −2.52	<0.001	

“*Interaction P” represents the significance of differences between subgroup*.

The results of the ESS scores in moderate OSA are summarized in Table 2 and Supplementary Figure 5. Meta-regression demonstrated that the efficacy of CPAP therapy was significantly associated with ESS score (*P* = 0.005), adherence (*P* <0.001), treatment duration (*P* = 0.009), and trial design (*P* = 0.001) after adjustment for age, BMI, and AHI. Subgroup analysis revealed significant differences according to the ESS score [ <11: WMD = −1.42, 95% CI (−1.57, −1.27) vs. ≥11: WMD = −2.22, 95% CI (−2.30, −2.13)], adherence [<4 h: WMD = −2.01, 95% CI (−2.09, −1.94) vs. ≥4 h: WMD = −2.59, 95% CI (−2.98, −2.20)], treatment duration [<3 months: WMD = −2.57, 95% CI (−2.98, −2.16) vs. ≥3 months: WMD = −2.02, 95% CI (−2.09, −1.94)], and trial design [parallel: WMD = −2.24, 95% CI (−2.32, −2.16) vs. crossover: WMD = −1.14, 95% CI (−1.30, −0.97)].

The results of meta-regression and subgroup analysis of ESS scores in severe OSA are summarized in Table 2 and Supplementary Figure 6. Meta-regression demonstrated that the efficacy of CPAP therapy was significantly associated with BMI (*P* = 0.0*2*8), ESS score (*P* = 0.001), and adherence (*P* = 0.047) after adjustment for age, BMI, and AHI. Subgroup analysis indicated significant differences according to BMI [<30: WMD = −1.17, 95% CI (−1.44, −0.90) vs. ≥30: WMD = −3.08, 95% CI (−3.24, −2.92)], ESS score [<11: WMD = −1.3, 95% CI (−1.55, −1.06) vs. ≥11: WMD = −3.22, 95% CI (−3.39, −3.05)], adherence [<5 h: WMD = −0.47, 95% CI (−1.63, −0.68) vs. ≥5 h: WMD = −2.12, 95% CI (−2.29, −1.96)], and treatment duration [<3 months: WMD = −2.34, 95% CI (−2.57, −2.20) vs. ≥3 months: WMD = −2.84, 95% CI (−3.05, −2.63)].

### Meta-Regression and Subgroup Analysis of Heterogeneity in the Change in Objective EDS

As shown in Table 3 and Supplementary Figure 7, meta-regression revealed that the AHI explained the heterogeneity of the change in MSLT very well (*P* = 0.004). Subgroup analysis based on OSA severity indicated that in mild, moderate, and severe OSA, the changes in MSLT were −0.23 min (95% CI: −1.11, 0.66), *I*^2^ = 0%, 1.02 min (95% CI: 0.55, 1.49), *I*^2^ = 28.1%, and 2.01 min (95% CI: 1.48, 2.54), *I*^2^ = 0%, respectively. These results indicated no significant effects of CPAP in mild OSA, whereas significant effects were observed in moderate and severe OSA, particularly the latter.

**Table 3:** Meta-regression and subgroup analysis for obejective excessive daytime sleepiness (EDS) based on Basic characteristics of patients and studies in different severity of OSA.

**Variables**	**Numbers of study**	**Subgroup analysis**			**Meta regression**
		**WMD**	**95% CI**	***P*-Value**	**Interaction *P***
**MSLT**	8				
OSA severity					
Mild	4	−0.23	−1.11,0.66	0.615	0.004
Moderate	2	1.02	0.55,1.49	<0.001	
Severe	2	2.01	1.48,2.54	<0.001	
**MWT**					
Baseline Age					
<50	2	2	1.61,2.40	<0.001	0.022
≥50	4	1.18	0.78,1.58	<0.001	
Baseline BMI					
<33	4	1.57	1.29,1.85	<0.001	0.003
≥33	2	15.34	9.49,21.20	<0.001	

“*Interaction P” represents the significance of differences between subgroup*.

As shown in Table 3 and Supplementary Figures 8, 9, meta-regression to assess the sources of heterogeneity revealed that age (*P* = 0.022) and BMI (*P* = 0.003) were the factors responsible for the change in MWT. Subgroup analysis indicated that patients <50 years of age [<50: WMD = 2.00, 95% CI (1.61, 2.40) vs. ≥50: WMD = 1.18, 95% CI (0.78, 1.58)] with BMI ≥33 kg/m^2^ [<33: WMD = 1.57, 95% CI (1.29, 1.85) vs. ≥33: WMD = 15.34, 95% CI (9.49, 21.20)] were more likely to show improvements in MWT.

### Publication Bias and Sensitivity Analysis

Begg's test and Egger's test for the evaluation of publication bias in terms of the ESS score returned *P*-values of 0.973 and 0.477, respectively, thus indicating an absence of publication bias. Begg's funnel plot and egger's publication bias plot are shown in Supplementary Figures 1, 2. Sensitivity analysis revealed that the overall effect size did not change when either study was removed. Therefore, the results of our meta-analysis were considered stable.

## Discussion

In this study, first, we conducted a meta-analysis of the efficacy of CPAP treatment, compared with placebo, in ameliorating EDS in patients with OSA. In total, 41 RCTs in 7,332 patients were included in our meta-analysis. A total of 40 studies reported ESS, demonstrating clinically significantly lower subjective sleepiness, by 2.14 points, among participants undergoing CPAP therapy than controls. In mild, moderate, and severe OSA, the ESS scores were 1.29 points, 2.03 points, and 2.58 points lower than those in controls, respectively. The above conclusions were consistent with the results reported by Marshall et al. (8) and Patil et al. (9). Second, eight studies reported MSLT and demonstrated a clinically significant prolongation of objective sleepiness by 1.23 min with CPAP therapy; however, this effect was not observed in the meta-analyses by Marshall et al. (8)and Patil et al. (9). This inconsistent result might be associated with the discordant severity of OSA among the patients enrolled. Therefore, we conducted a subgroup analysis and found that patients with mild OSA did not show improvements in MSLT with CPAP therapy, thus further confirming our hypothesis. Finally, six studies reported MWT, and the results demonstrated a clinically significant increase in objective wakefulness by 1.6 min. Subgroup analysis indicated that patients <50 years of age with a BMI of >33 kg/m^2^ may benefit more from CPAP therapy.

The efficacy of CPAP therapy in ameliorating subjective sleepiness was highly heterogeneous, at 95%. To explore the source of this heterogeneity, we performed a subgroup analysis based on OSA severity. The heterogeneity remained high in patients with mild, moderate, and severe OSA, at 53.2, 95.5, and 95.7%, respectively. Therefore, we conducted further subgroup analysis and meta-regression based on the characteristics of the study and mild, moderate, or severe OSA status. In mild OSA, heterogeneity in ESS was associated with baseline age, BMI, ESS score, compliance, and duration of treatment; patients ≥50 years of age with a BMI <30 kg/m^2^, ESS scores <11, adherence <3 h/night, and treatment duration <2 months showed no significant improvements. In moderate OSA, heterogeneity in ESS was associated with the baseline ESS score, compliance, duration of treatment, and type of trial design; patients with ESS scores ≥11, adherence ≥4 h/night, treatment duration <3 months, and parallel RCTs showed significant improvement. In severe OSA, heterogeneity in ESS was associated with baseline BMI, ESS score, compliance, and duration of treatment; patients with BMI ≥30 kg/m^2^, ESS scores ≥11, adherence ≥5 h/night, and treatment duration ≥3 months showed greater changes in ESS. Overall, our results extend the previous meta-analysis reported by Marshall et al. (8) and Patil et al. (9) by meta-regression analyzing that treatment of OSA with CPAP results in more significant efficacy in EDS in patients who were sleepier and younger at baseline and who had higher BMI and good adherence.

Our subgroup analysis based on the basic clinical characteristics of patients revealed the following findings. Younger patients with OSA may be more likely to benefit from CPAP therapy. Patients ≥50 years of age with mild OSA may not benefit from CPAP therapy for subjective sleepiness, and patients <50 years of age show more significant improvements in objective wakefulness. Older patients appear to respond less well to CPAP therapy—an interesting finding that we speculate might be due to drowsiness in older patients, under the influence of potential confounding factors beyond OSA, such as sleep rhythm disorders, cerebrovascular diseases, and concomitant use of sleeping medications (55). Therefore, the underlying cause of drowsiness in patients with OSA with EDS must be comprehensively evaluated before CPAP therapy. In addition, obese patients showed a more favorable response to CPAP treatment than nonobese patients. Some studies have shown that BMI has no effect on the efficacy of CPAP therapy (56, 57). However, others have concluded that BMI does affect the efficacy of CPAP therapy (58, 59), in agreement with our results. Tangugsorn's research (60) based on cephalometric analyses has shown that nonobese patients with OSA tend to have craniofacial bone structure malformations, whereas obese patients with OSA primarily show abnormalities in upper airway soft tissue. This finding also suggests that different treatment options should be provided for obese and nonobese patients with OSA. First, obese patients may respond well to CPAP therapy, in contrast to nonobese patients, who may benefit more from surgical treatment. Second, obesity may contribute to daytime sleepiness by increasing the production of pro-inflammatory somatic adipose tissue-derived cytokines, such as interleukin (IL)-6 and plasminogen activator inhibitor (PAI)-1 (61). CPAP therapy effectively decreases the inflammatory response in obese patients with OSA, thereby improving EDS. Moreover, patients with low BMI may not respond well to CPAP therapy. A study comparing obese OSA with nonobese OSA has indicated that nonobese OSA is associated with a lower arousal threshold for airway stenosis, which may limit CPAP resistance and CPAP adherence, thus leading to poor outcomes (62). The combination of these factors may result in obese patients particularly benefitting from CPAP therapy to improve daytime function, such as EDS. Third, the baseline ESS score is a sensitive predictor of ameliorated subjective sleepiness in patients with OSA treated with CPAP. Regardless of the OSA severity, patients with a higher baseline ESS score were more likely to benefit from subjective sleepiness, whereas ESS scores did not predict the change in objective sleepiness or objective wakefulness. The MSLT, MMT test, and questionnaire (ESS) measured different objective and subjective aspects of sleepiness. Huang et al. (62) have found that, among untreated patients with OSA, the increase in subjective sleepiness is not significantly associated with a decrease in objective sleep latency and inability to remain awake. This result supports the findings of our meta-analysis, in which baseline ESS scores did not predict amelioration of objective sleepiness in patients with OSA treated with CPAP. Furthermore, although CPAP therapy significantly improves EDS, individuals do not respond equally to treatment. Baseline age, BMI, and ESS scores as clinical markers have potential clinical utility for identifying patients with OSA who are more likely to show EDS improvement. Further studies should be conducted to examine the links between these clinical markers and CPAP response and their underlying mechanisms.

On the basis of the subgroup analysis of CPAP compliance, treatment duration, and study characteristics, we obtained the following findings. Despite a consensus in which good CPAP adherence is defined as 4 h/night over 5 days/week, the optimal level of CPAP use appears to vary depending on the symptoms and the target treatment. Masa et al. (63) have demonstrated that subjective sleepiness symptoms normalize when compliance reaches 4 h/night, whereas Weaver et al. (64) has found that more than 5 h is required in severe OSA. How many hours of CPAP per night can significantly ameliorate daytime sleepiness at different levels of OSA severity? Our results demonstrated that CPAP compliance in ameliorating subjective sleepiness showed a dose-response effect depending on OSA severity. For instance, in mild OSA, CPAP use reaching 3 h/night can be beneficial, whereas for moderate or severe OSA, compliance should be increased to 4 or 5 h/night, respectively, to achieve better effects. Our results suggest that the traditional CPAP use threshold of 4 h/night does not perfectly normalize EDS. Therefore, we recommended that the minimum threshold for CPAP compliance be stratified according to OSA severity to control daytime symptoms in future clinical work. However, adherence to CPAP varies widely among individuals. In prior studies, 29%−83% of patients have been reported to use CPAP for <4 h/night (65). The effectiveness of CPAP therapy is often limited by suboptimal adherence, and strategies that can be implemented in clinical work to optimize adherence will be crucial for future research. In terms of treatment duration, mild to moderate OSA showed a good curative effect within 3 months, and severe OSA showed a good curative effect after more than 3 months. These findings suggested that the duration of CPAP therapy should be extended to more than 3 months in clinical practice for severe OSA. Finally, the trials in our meta-analysis included both parallel and crossover RCTs. Compared with parallel RCTs, crossover RCTs have the advantage of eliminating individual differences and improving statistical accuracy. Our subgroup analysis demonstrated that CPAP therapy significantly decreased subjective and objective sleepiness in both parallel and crossover RCTs, thus providing convincing results.

Our review has the following strengths: we included RCTs with a large sample size, which was high quality evaluated by the JADAD scale and Cochrane risk bias assessment tool, indicating that the quality of evidence provided by our meta-analysis is high. However, there are a few studies with reporting bias and attrition bias. Furthermore, the lack of detail on the randomization methods used in some studies leads to a high/unclear risk of bias, which may reflect reporting problems rather than genuine methodological flaws. We reported the first evidence that some subtypes with mild OSA may not achieve subjective sleepiness benefits from CPAP therapy, and CPAP therapy can ameliorate objective sleepiness in patients with moderate-severe OSA. We identified no publication bias, and the results of sensitivity analysis were stable. Although the heterogeneity was high, we identified the source of the heterogeneity and predictors of CPAP therapy efficacy through meta-regression and subgroup analysis. Our review also has several limitations, as follows: our meta-analysis was not registered, and there may be a small bias, but we still strictly followed the steps of systematic evaluation. Although tests for publication bias did not show statistical significance, it is always possible that there are unpublished negative trials that lead to overestimates of the efficacy of CPAP. We restricted eligibility to studies in English only, which potentially led to language bias. Meta-analysis of individual patient data (IPD meta-analysis) can make the results more realistic, but due to the large number of studies involved, obtaining original data would be challenging, so we did not perform an IPD meta-analysis. Some studies included OSA with other diseases (e.g., stroke, diabetes, or hypertension), thus potentially interfering with the results of our meta-analysis, although we performed meta-regression to correct for confounding factors. The RCTs included in our review included both parallel and crossover trial designs; therefore, we conducted a subgroup analysis according to trial design type and found that patients were more likely to benefit from CPAP therapy in parallel RCTs. The combination of parallel and crossover analyses is considered statistically irregular, given the different statistical methods used to calculate the standard error; this aspect remains a potential weakness of the review.

The American Academy of Sleep Medicine (AASM) practice parameters (66) recommended CPAP as the first-line treatment for moderate to severe OSA, and CPAP therapy may also be attempted for mild OSA with clinical symptoms. Our review could provide evidence in support of previously published AASM practice parameters regarding the efficacy of CPAP therapy for OSA. However, OSA is considered a complex and heterogeneous sleep disorder (67). Diagnosis, severity assessment, and treatment of OSA still often rely on a single indicator, the AHI, which is highly flawed (68). Previous studies (69, 70) have demonstrated that the severity of daytime sleepiness is poorly correlated with OSA severity. Some patients still have residual daytime sleepiness after CPAP therapy despite normalization of AHI. Other indicators must urgently be explored for OSA stratification, to improve understanding of the genetic and biological mechanisms and to identify OSA subtypes most suitable for CPAP therapy. Therefore, we conducted this meta-analysis and found that patients who were drowsy, obese, younger, and compliant appeared to be more likely to experience improvement in EDS after CPAP therapy. Overall, our meta-analysis appears to have captured prognostic heterogeneity, owing to some clinical characteristics of patients with OSA, such as age, BMI, EDS, and compliance. However, whether these represent true subtypes remains to be identified in future studies. The pattern of response to OSA treatment varies depending on the initial clinical subtype and CPAP adherence (71, 72). Our results suggest that the proposed subtype classification provides prognostic information related to CPAP treatment outcomes for daytime sleepiness that existing clinical criteria do not provide alone.

In summary, our meta-analysis highlight that age, BMI, ESS scores, and CPAP adherence are useful predictors to identify patients with OSA likely to respond to CPAP with a reduction in EDS. These results may help sleep specialists identify patients with OSA who have an advantage in CPAP therapy for EDS. Notably, this finding suggests that the identification of patients using clinical predictors may provide a new paradigm for understanding the treatment expectations of patients with OSA, thus facilitating the early identification and prognosis of OSA. Future research should focus on the identification of more effective clinical markers and explore the underlying mechanisms by which these predictors mediate CPAP response.

## Data Availability Statement

The original contributions presented in the study are included in the article/Supplementary Material, further inquiries can be directed to the corresponding author.

## Author Contributions

JW participated in data collection and verification. SC helped perform the analysis with constructive discussions. ZL performed the data analyses and wrote the manuscript. RC contributed to the conception of the study. All authors contributed to the article and approved the submitted version.

## Funding

This study was funded by the Natural Science Foundation of China (Grant numbers: NSFC81770085 and 82070095).

## Conflict of Interest

The authors declare that the research was conducted in the absence of any commercial or financial relationships that could be construed as a potential conflict of interest.

## Publisher's Note

All claims expressed in this article are solely those of the authors and do not necessarily represent those of their affiliated organizations, or those of the publisher, the editors and the reviewers. Any product that may be evaluated in this article, or claim that may be made by its manufacturer, is not guaranteed or endorsed by the publisher.
