# Supplementary material for: Predictors of the Efficacy for Daytime Sleepiness in Patients With Obstructive Sleep Apnea With Continual Positive Airway Pressure Therapy: A Meta-Analysis of Randomized Controlled Trials
Source: Front Neurol. 2022 Jun 27;13:911996. doi: 10.3389/fneur.2022.911996 (PMC9271709; doi:10.3389/fneur.2022.911996)
Supplement: Supplementary file 1 [file Data_Sheet_1.pdf]

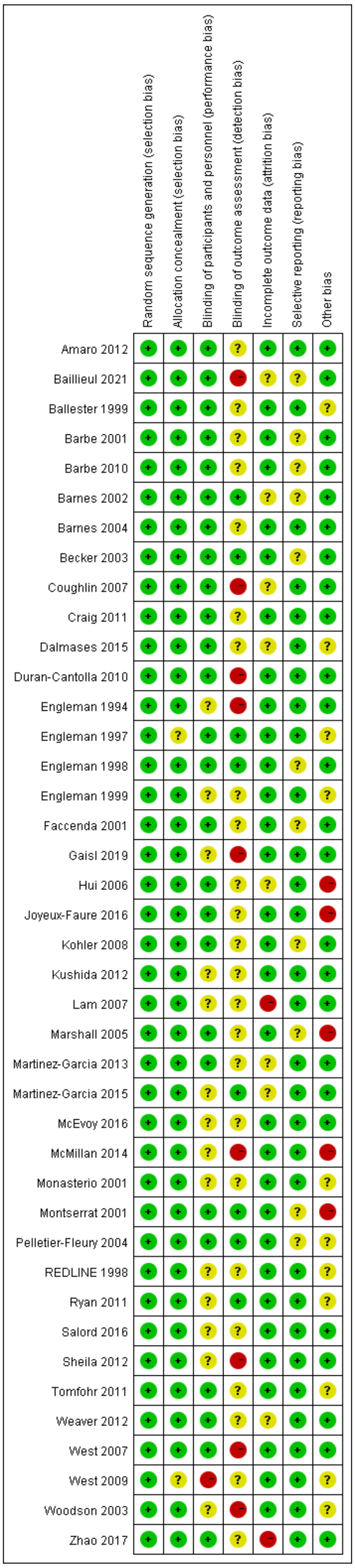

Supplementary figure 1: Risk of bias summary：The Cochrane risk bias assessment tool was used to evaluate bias from seven key sources: 1. Random sequence generation; 2. Allocation concealment; 3. Blindness of subjects and researchers; 4. Blindness of outcome evaluation; 5. Incomplete data; 6. Selective reporting of results; 7. Other biases. Green represents low risk, yellow medium risk, and red high risk.

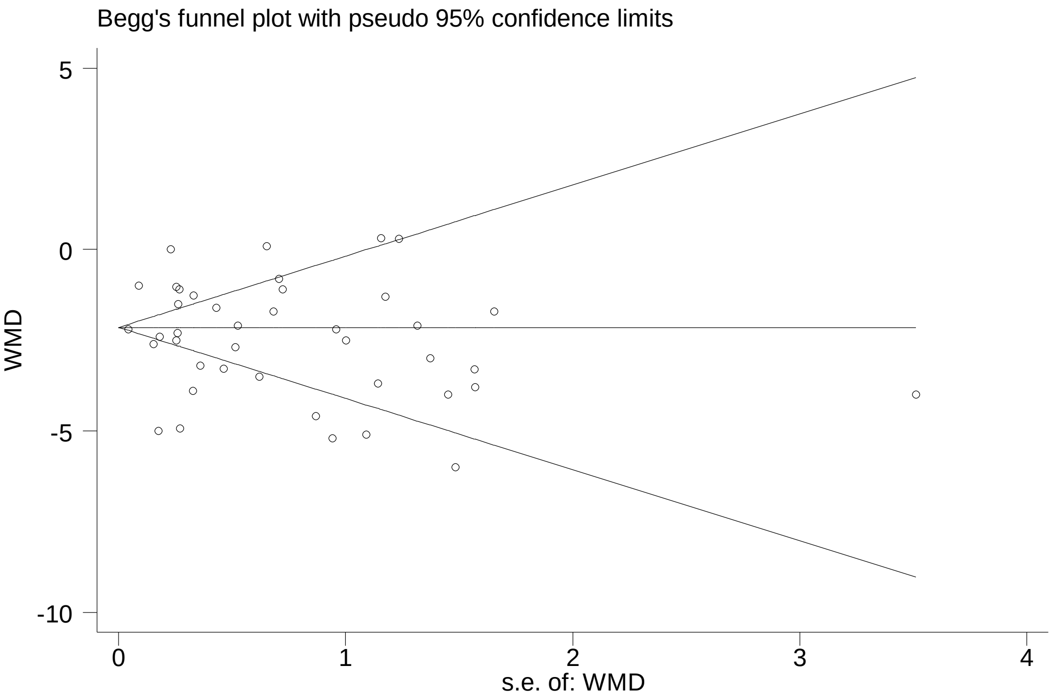

Supplementary figure 2: Begg’s funnel plot

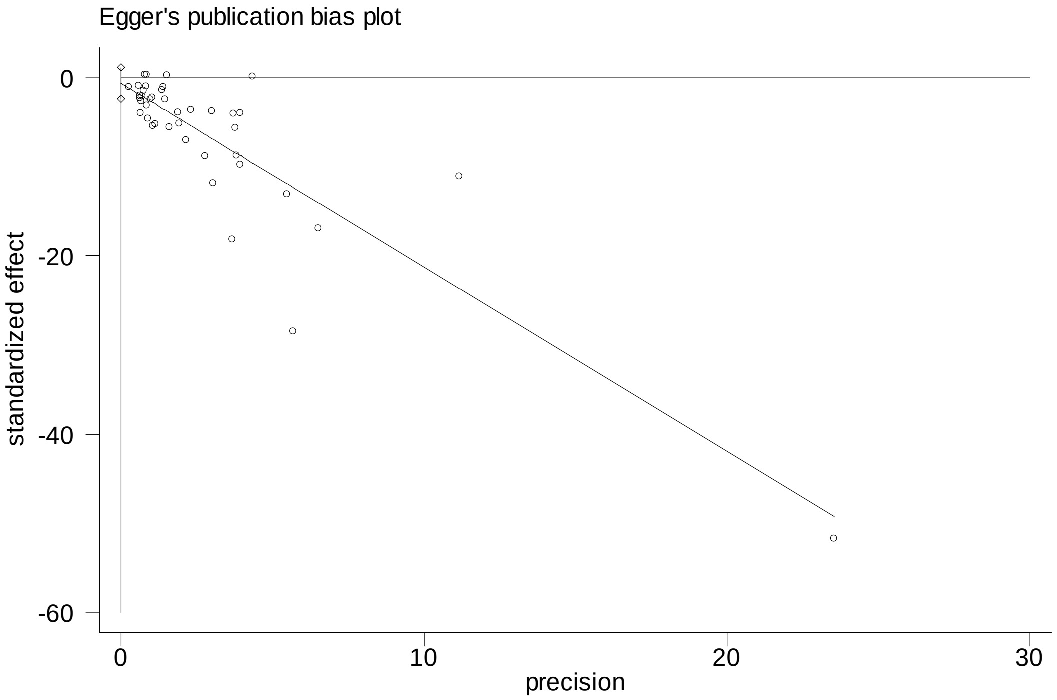

Supplementary figure 3: Egger’s publication bias plot

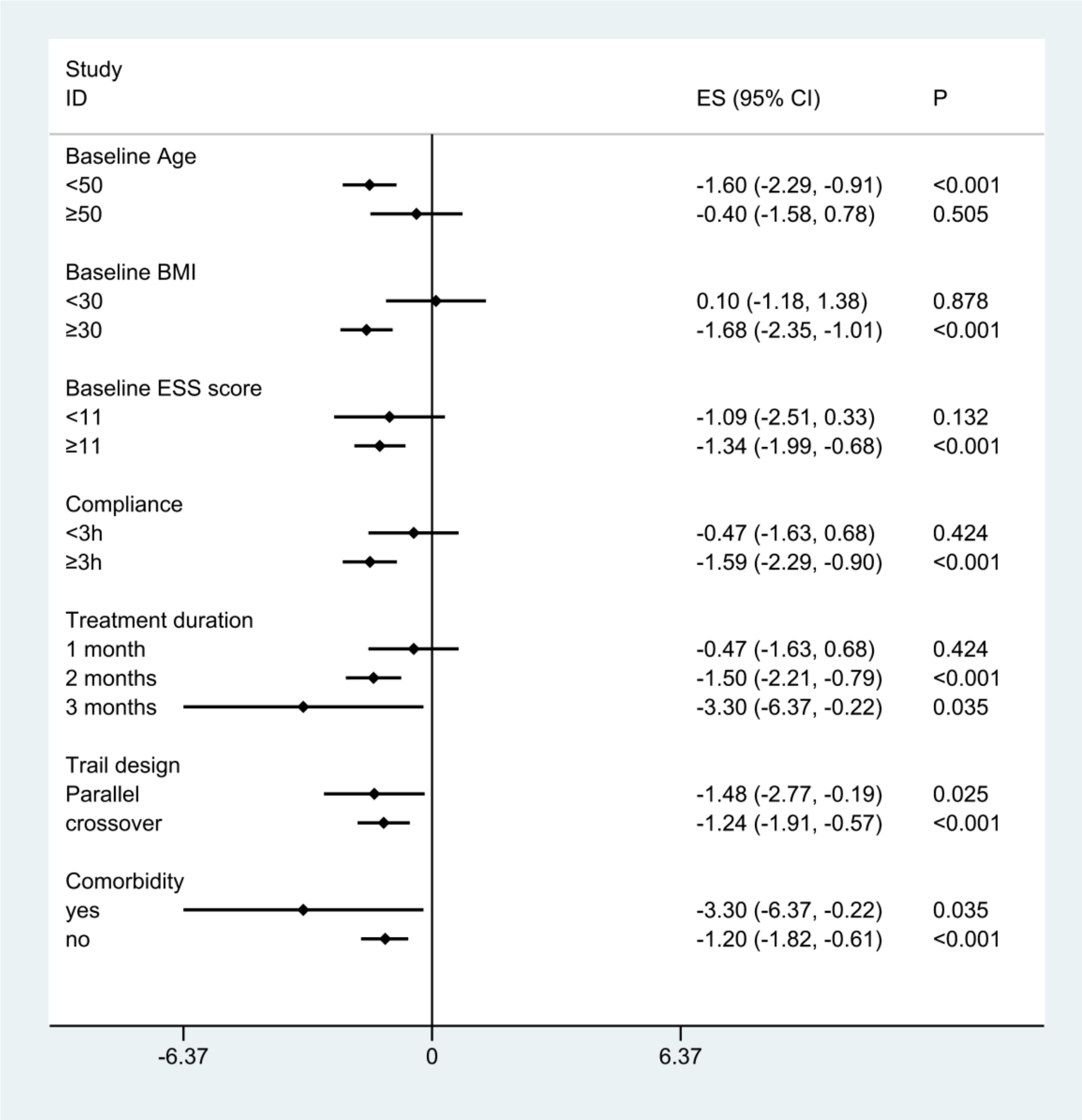

Supplementary figure 4: Subgroup analysis of ESS scores in mild OSA

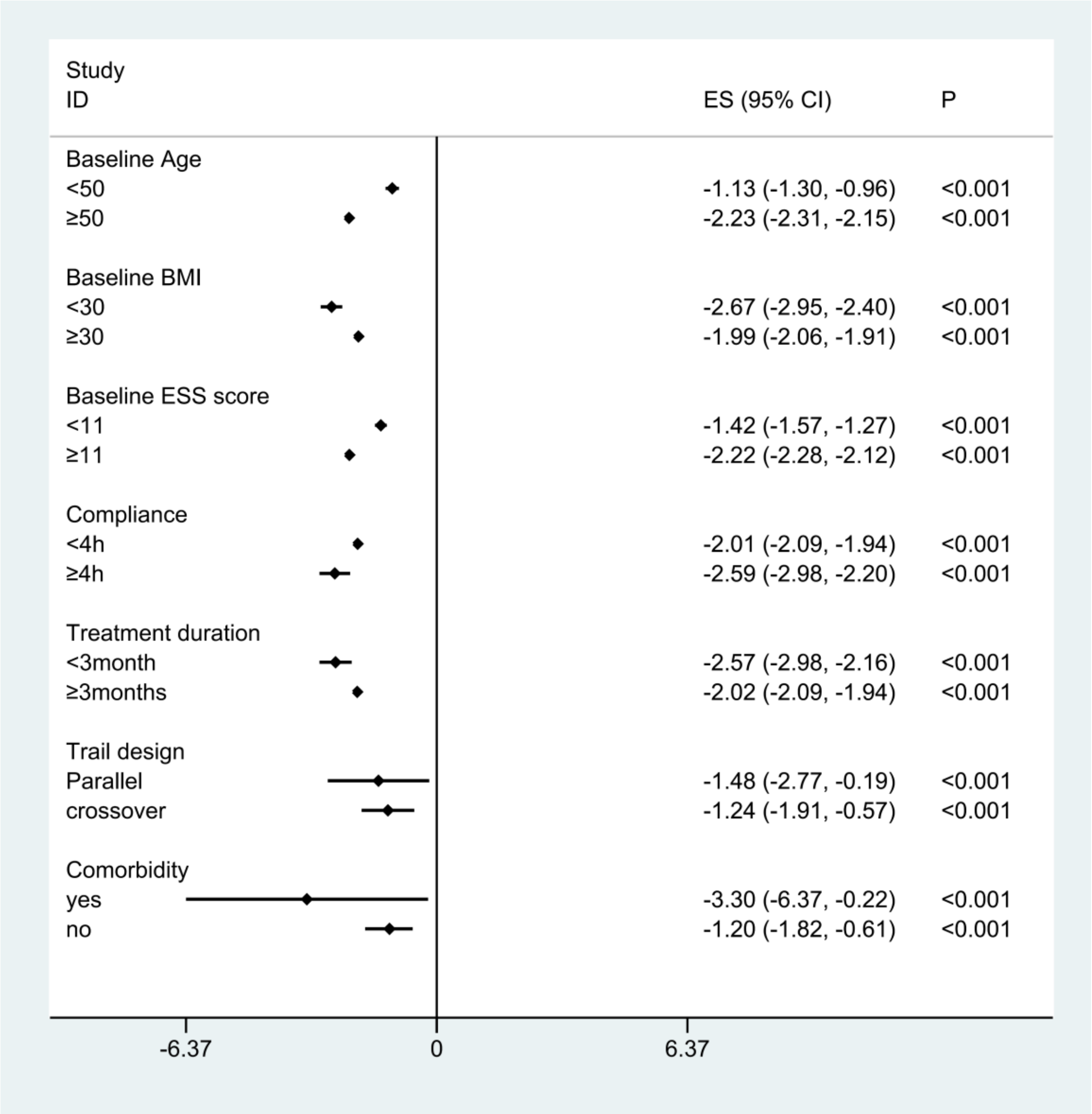

Supplementary figure 5: Subgroup analysis of ESS scores in moderate OSA

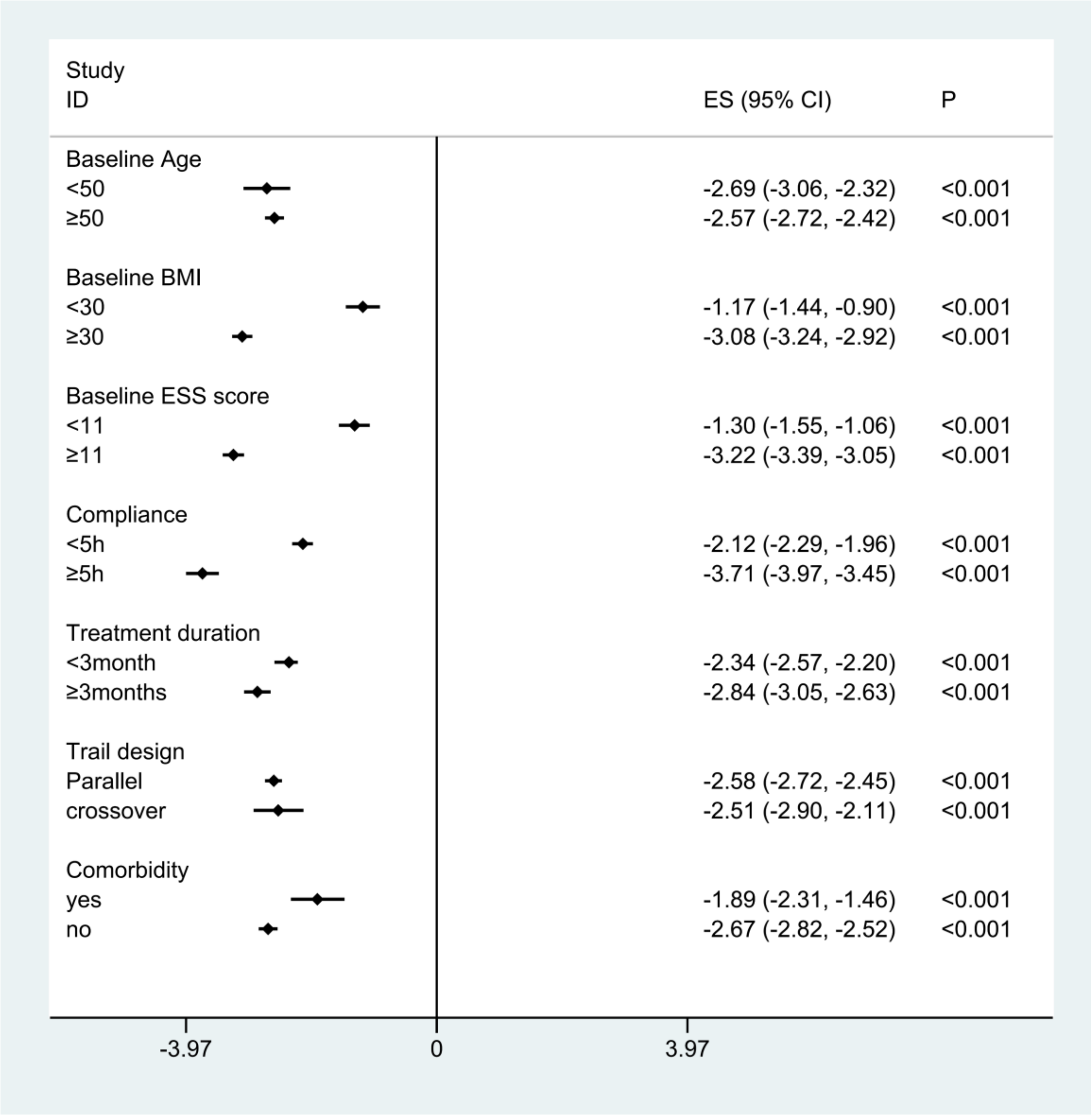

Supplementary figure 6: Subgroup analysis of ESS scores in severe OSA

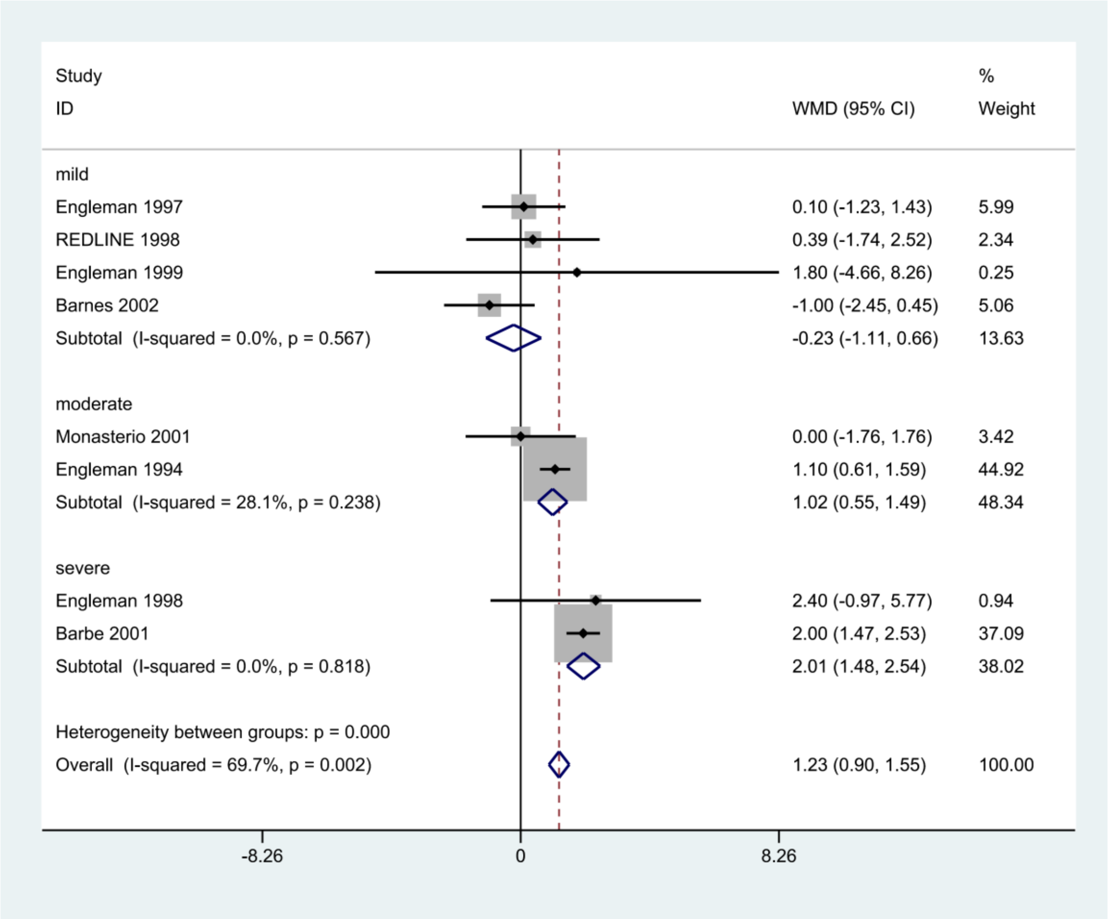

Supplementary figure 7: Subgroup analysis of MSLT based on OSA severity.

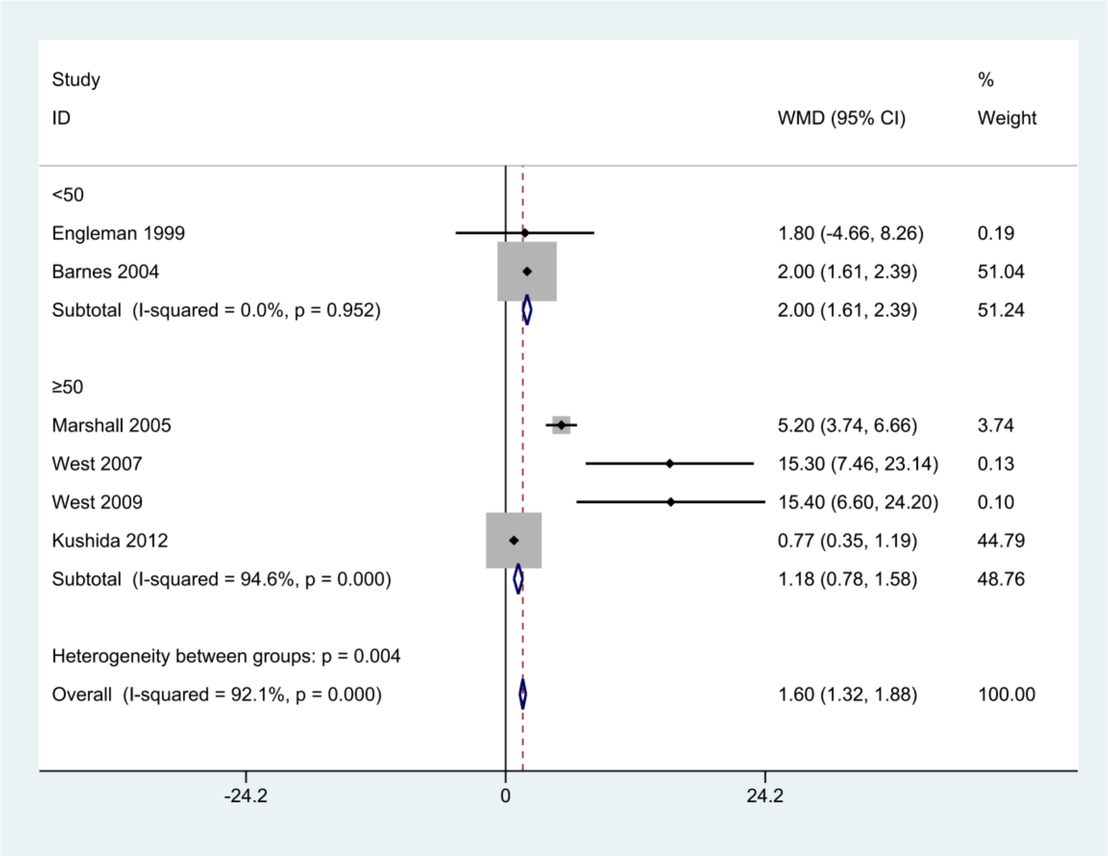

Supplementary figure 8: Subgroup analysis of MWT based on age.

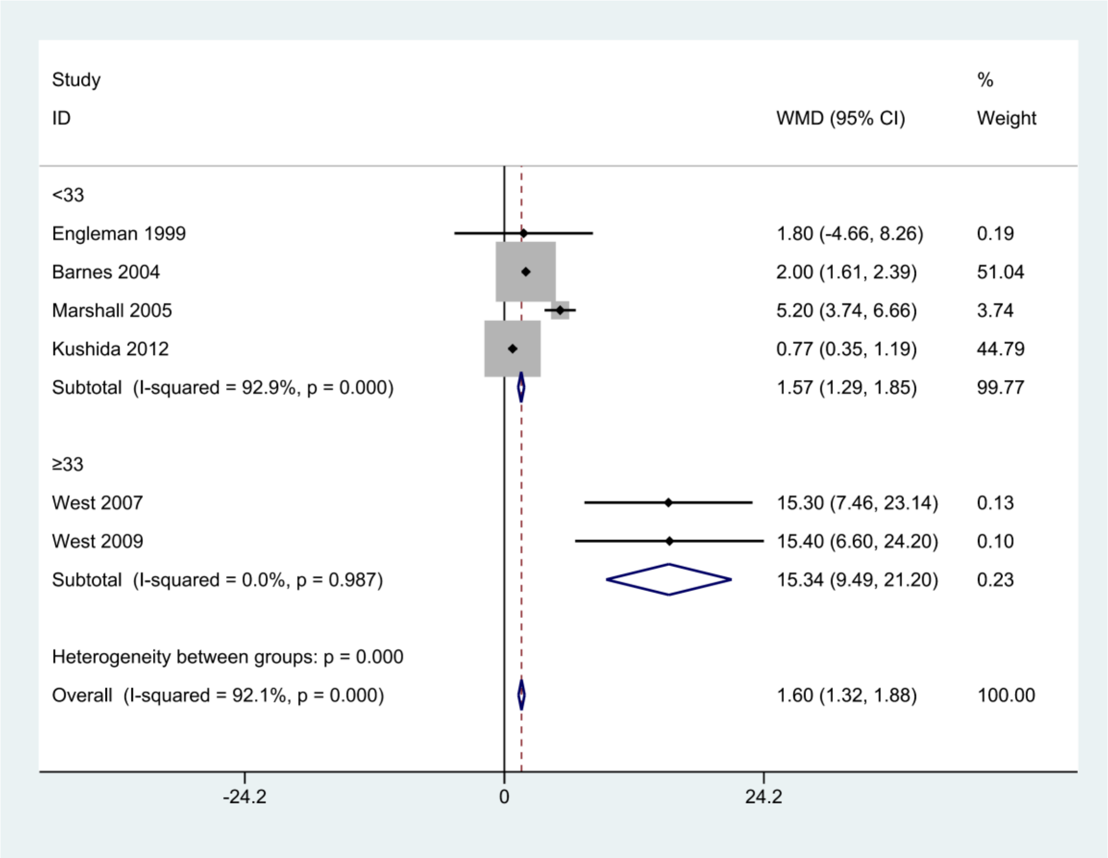

Supplementary figure 9: Subgroup analysis of MWT based on BMI.
